# Work-family conflict and its related factors among emergency department physicians in China: A national cross-sectional study

**DOI:** 10.3389/fpubh.2023.1092025

**Published:** 2023-03-20

**Authors:** Shijiao Yan, Changjun Li, Jiali Zhang, Yafei Wu, Mengge Tian, Li Liu, Xuan Zhou, Jianwei Zheng, Nan Jiang

**Affiliations:** ^1^Department of Emergency Medicine, Hunan Provincial Key Laboratory of Emergency and Critical Care Metabolomics, Hunan Provincial Institute of Emergency Medicine, Hunan Provincial People's Hospital/The First Affiliated Hospital, Hunan Normal University, Changsha, China; ^2^School of Public Health, Hainan Medical University, Haikou, China; ^3^Department of Neurology, The Central Hospital of Wuhan, Tongji Medical College, Huazhong University of Science and Technology, Wuhan, China; ^4^Department of Social Medicine and Health Management, School of Public Health, Tongji Medical College, Huazhong University of Science and Technology, Wuhan, China; ^5^Office of Academic Research, The Central Hospital of Wuhan, Tongji Medical College, Huazhong University of Science and Technology, Wuhan, China; ^6^Department of Anesthesiology, Tongji Hospital, Tongji Medical College, Huazhong University of Science and Technology, Wuhan, China; ^7^Department of Biliary-Pancreatic Surgery, Tongji Hospital, Tongji Medical College, Huazhong University of Science and Technology, Wuhan, China

**Keywords:** work-family conflict, emergency department physicians, related factors, China, cross-sectional

## Abstract

**Background:**

Work-family conflict is common among emergency department physicians. Identifying the factors associated with work-family conflict is key to reducing its negative impact on mental health and work attitudes. However, the work-family conflict of Chinese emergency department physicians and the related factors have been scarcely studied.

**Objective:**

This study aimed to investigate the current status and related factors of work-family conflict among Chinese emergency department physicians.

**Methods:**

A national cross-sectional study was conducted among emergency department physicians in China from June 2018 to August 2018. A standard questionnaire was used to investigate the demographic characteristics, work-related factors, and work-family conflict of emergency department physicians. The generalized linear regression analysis was used to identify the related factors of work-family conflict.

**Results:**

A total of 10,457 licensed emergency department physicians participated in the study. The average score of work-family conflict among the enrolled emergency department physicians was 19.27 ± 3.94, and the prevalence of high levels of work-family conflict was 69.19%. The multivariable regression analysis showed that emergency physicians who were female (linear regression coefficient, −0.25; SE, 0.08; *P* = 0.002), older than 40 years (linear regression coefficient,−0.53; SE, 0.14; *P* < 0.001), and earning more than 4,000 CNY per month (e.g., 4,001~6,000 vs. ≤4,000 CNY: linear regression coefficient, −0.17; SE, 0.09; *P* = 0.04) had lower work-family conflicts. However, emergency department physicians who were married (linear regression coefficient, 0.37; SE, 0.11; *P* < 0.001), highly educated (linear regression coefficient, 0.46; SE, 0.10; *P* < 0.001), had a high technical title (e.g., intermediate vs. junior technical title: linear regression coefficient, 0.61; SE, 0.09; *P* < 0.001), worked in a high-grade hospital (e.g., tertiary hospital vs. emergency center: linear regression coefficient, 0.38; SE, 0.11; *P* < 0.001), had a higher frequency of night shifts (e.g., 6~10 night shifts per month vs. 0~5 night shifts per month: linear regression coefficient, 0.43; SE, 0.10; *P* < 0.001), self-perceived shortage of physicians in the department (linear regression coefficient, 2.22; SE, 0.08; *P* < 0.001), and experienced verbal abuse (linear regression coefficient, 1.48; SE, 0.10; *P* < 0.001) and physical violence (linear regression coefficient, 0.84; SE, 0.08; *P* < 0.001) in the workplace had higher work-family conflict scores.

**Conclusion:**

Most emergency department physicians in China experience a high-level work-family conflict. Hospital administrations are recommended to develop family-friendly workplace policies, establish a scientific shift system, and keep the number of emergency department physicians to meet the demand to reduce work-family conflict.

## Introduction

Work-family conflict is an inter-role conflict that results from the incompatibility of role pressures between work and family domains (1). According to scarcity theory, personal resources, such as time and energy, are limited. The devotion of more resources to work role will inevitably lead to a reduction in the devotion of resources to family role (2, 3). Emergency department physicians are the first line of defense in hospitals (4). In addition to work at a fast pace and with high intensity (5, 6), they are required to respond to unforeseen medical situations around-the-clock (7, 8), making them devote more resources to work role and prone to work-family conflict. The existing studies also reported that the work-family conflict among emergency department physicians was significantly higher than that of physicians in other departments (4, 9).

The work-family conflict has a series of negative impacts on both physicians and hospitals. At the individual level, work-family conflict has been reported to be related to psychological distress (10). For example, work-family conflict was found to be associated with mental stress among German physicians (11) and anxiety symptoms among Chinese doctors (12). A prospective study in the United States found a significant relationship between work-family conflict and a higher prevalence of depressive symptoms among physicians (13). Furthermore, conflict between work and family is known to increase the risk of both acute and chronic physical health issues (14). At the hospital level, work-family conflict positively correlates with job burnout (15) and turnover intention (16), which can reduce physicians' productivity and increase hospital operating costs (17, 18). Given these unfavorable outcomes, it is necessary to identify the related factors of work-family conflict among emergency department physicians.

However, most of the studies on physicians' work-family conflict have mainly focused on its negative consequences (11, 19–22), and few studies have explored the factors associated with work-family conflict (23). Moreover, there is a lack of research on the related factors of work-family conflict among emergency department physicians. In China, there is a severe shortage of emergency department physicians, making them more vulnerable to work-family conflict than in other countries. Therefore, we aimed to conduct a national survey in China to explore the current status and related factors of work-family conflict among emergency department physicians, so as to provide a scientific basis for the hospital administrations to formulate interventions.

## Methods

### Ethics statement

The study was approved by the Research Ethics Committee in Hainan Medical University (approval number: HYLL-2018-035). All participants volunteered to take part in this survey and all private information of them was kept confidential.

### Participants and data collection

A nationwide cross-sectional study of emergency department physicians was conducted in China from July 2018 to August 2018 under the coordination of the Medical Administration Bureau of the National Health Commission. Data were collected through a widely used online survey platform, Questionnaire Star (website: https://www.wjx.cn). The link of electronic questionnaire was posted on the emergency department physicians' work platform of the prehospital emergency facility configuration monitoring department. Emergency department physicians from 2,965 public hospitals that provided pre-hospital emergency care in 31 provinces could click the link. Survey link was re-posted to the work platform every 7 days during the survey period. All respondents were required to complete an informed consent form before answering the questionnaire. Also, each questionnaire could only be submitted if all questions were answered, so there was no missing data for each variable. In this study, 15,288 emergency department physicians clicked the link of the electronic questionnaire, and 10,457 submitted it. The completion rate was 68.4%.

### Measurements

The questionnaires covered demographic characteristics, work-related factors, and work-family conflict. Demographic characteristics included gender, age, educational level, and marital status. Work-related factors included technical titles, monthly income, years of service, frequency of night-shift per month, and self-perceived shortage of physicians in the emergency department. The question “Do you think the number of physicians in the emergency department meets the demands of daily work?” was used to measure the perceived shortage of physicians in the emergency department. If the respondents answered that the number of physicians could meet daily needs, it represented no self-perceived shortage of physicians; on the contrary, it represented a self-perceived shortage of physicians.

Work-family conflict was measured by the 5-item Work-family Conflict scale developed by Netemeyer et al. (24). The items were rated on a 5-point Likert scale, ranging from 1 (strongly disagree) to 5 (strongly agree). Higher scores indicated higher levels of work-family conflict. Furthermore, the average scores of the five items were re-classified into three categories, those who scored <2.5 were re-classified into the “low work-family conflict” group, those who scored between 2.5 and 3.6 were re-classified into the “medium work-family conflict” group, and those who scored more than 3.6 were re-classified into the “high work-family conflict” group (4). In this study, Cronbach's α for the scale was 0.934.

### Statistical analysis

SPSS 25.0 for Windows was used to perform data analyses. In descriptive analyses, continuous variables were represented by mean and standard deviation (SD), while categorical variables were represented by frequency and percentage. *T*-test and one-way ANOVA were performed to examine the differences of work-family conflict scores among groups with diverse characteristics. Spearman correlations were used to test multicollinearity among independent variables. We started with univariable analysis to screen for candidate variables associated with work-family conflict using a cutoff value of *P* < 0.1. A generalized linear regression model was used to identify the related factors of work-family conflict. All comparisons were two-tailed and the significance threshold was *P* < 0.05.

## Results

The basic characteristics of the participants are shown in Table 1. Among 10,457 emergency department physicians, 72.98% were males. Nearly two-thirds of the participants were younger than 40 years old. Most of them were married, accounting for 84.42%. About five-sixths of participants obtained a bachelor's degree. Nearly half of the participants had junior technical titles, worked in secondary hospitals, engaged in emergency work for 6 years or more, and worked 6~10 night shifts per month. Only 29% of physicians earned more than 6,000 CNY per month. Approximately 70% of physicians perceived a shortage of emergency department physicians. 81.81 and 27.63% of physicians experienced verbal abuse and physical violence in the workplace, respectively.

**Table 1:** Participants' characteristics and their associations with work-family conflict.

**Variables**	***n***	**%**	**WFC (*M* ±SD)**	***F/t***	***P***
**Total**	10,457		19.27 ± 3.94		
**Gender**					
Male	7,632	72.98	19.48 ± 3.89	8.81	<0.001
Female	2,825	27.02	18.71 ± 4.02		
**Age (year)**					
≤29	1,925	18.41	18.63 ± 4.19	34.56	<0.001
30~34	2,728	26.09	19.53 ± 3.91		
35~39	2,463	23.55	19.72 ± 3.92		
≥40	3,341	31.95	19.09 ± 3.76		
**Marital status**					
Unmarried/widowed/divorced/separated	1,629	15.58	18.62 ± 4.21	−6.92	<0.001
Married	8,828	84.42	19.39 ± 3.88		
**Educational level**				−9.53	<0.001
Junior college degree or less	1,684	16.10	18.41 ± 4.06		
Bachelor degree and above	8,773	83.90	19.44 ± 3.90		
**Technical title**					
Junior	4,972	47.55	18.92 ± 4.11	42.26	<0.001
Intermediate	4,112	39.32	19.69 ± 3.78		
Senior	1,373	13.13	19.28 ± 3.68		
**Type of hospital**				17.34	<0.001
Emergency center	1,681	16.08	18.97 ± 4.11		
Primary	748	7.15	18.46 ± 4.18		
Secondary	4,442	42.48	19.39 ± 3.85		
Tertiary	3,586	34.29	19.44 ± 3.89		
**Monthly income (CNY)**					
≤4,000	3,862	36.93	19.24 ± 4.00	2.34	0.10
4,001~6,000	3,562	34.06	19.38 ± 3.84		
≥6,001	3,033	29.00	19.18 ± 3.97		
**Years of service**					
<1	1,448	13.85	18.51 ± 4.10	48.65	<0.001
1~5	3,965	37.92	19.12 ± 3.92		
≥6	5,044	48.24	19.61 ± 3.87		
**Frequency of night shift (per month)**					
0~5	2,033	19.44	18.09 ± 3.91	174.41	<0.001
6~10	5,633	53.87	19.24 ± 3.87		
≥11	2,791	26.69	20.20 ± 3.87		
**Self-perceived shortage of physicians**					
No	2,790	26.68	17.22 ± 3.97	−32.59	<0.001
Yes	7,667	73.32	20.02 ± 3.65		
**Verbal abuse in the workplace**				−24.77	<0.001
No	1,902	18.19	17.18 ± 4.15		
Yes	8,555	81.81	19.74 ± 3.74		
**Physical violence in the workplace**				−19.99	<0.001
No	7,568	72.37	18.81 ± 3.91		
Yes	2,889	27.63	20.47 ± 3.75		

WFC, work-family conflict.

The average score of work-family conflict among the enrolled emergency department physicians was 19.27 (SD = 3.94). Moreover, 7,235 participants (69.19%) were in the “high work-family conflict” group, 2,741 participants (26.21%) were in the “medium work-family conflict” group, and 481 participants (4.60%) were in the “low work-family conflict” group.

The univariate analysis results are shown in Table 1. There were significant differences in work-family conflict scores in gender, age, marital status, educational level, technical title, type of hospital, monthly income, years of service, frequency of night-shift per month, self-perceived shortage of physicians, verbal abuse and physical violence at workplace.

Based on the results of the Spearman correlation analysis (Supplementary Table S1), we excluded length of service from the multivariable analysis to minimize multicollinearity among independent variables. The findings for the multivariable analyses are presented in Table 2. Emergency physicians who were female (linear regression coefficient, −0.25; SE, 0.08; *P* = 0.002), older than 40 years (linear regression coefficient, −0.53; SE, 0.14; *P* < 0.001), and earning more than 4,000 CNY per month (e.g., 4,001~6,000 vs. ≤4,000 CNY: linear regression coefficient, −0.17; SE, 0.09; *P* = 0.04) suffered less work-family conflicts. However, emergency department physicians who were married (linear regression coefficient, 0.37; SE, 0.11; *P* < 0.001), highly educated (linear regression coefficient, 0.46; SE, 0.10; *P* < 0.001), and with a higher technical title (e.g., intermediate vs. junior technical title: linear regression coefficient, 0.61; SE, 0.09; *P* < 0.001) scored more points for work-family conflict. Physicians who worked in secondary hospital (linear regression coefficient, 0.33; SE, 0.10; *P* = 0.001) and tertiary hospital (linear regression coefficient, 0.38; SE, 0.11; *P* < 0.001) had higher work-family conflict scores compared to physicians working in emergency center. In addition, emergency department physicians with a high frequency of night shifts (e.g., 6~10 night shifts per month vs. 0~5 night shifts per month: linear regression coefficient, 0.43; SE, 0.10; *P* < 0.001), self-perceived shortage of physicians (linear regression coefficient, 2.22; SE, 0.08; *P* < 0.001), and experienced verbal abuse (linear regression coefficient, 1.48; SE, 0.10; *P* < 0.001) and physical violence (linear regression coefficient, 0.84; SE, 0.08; *P* < 0.001) at the workplace scored higher on work-family conflict.

**Table 2:** General linear regression analysis of related factors of work-family conflict.

**Variables**	**Coefficient**	**SE**	***t***	***P***	**95%CI**	
Constant	14.74	0.18	81.09	<0.001	14.38	15.09
**Gender**						
Male^*^	0.00	–	–	–	–	–
Female	−0.25	0.08	−3.12	0.002	−0.41	−0.09
**Age (year)**						
≤29^*^	0.00	–	–	–	–	–
30~34	0.14	0.12	1.20	0.23	−0.09	0.37
35~39	−0.05	0.13	−0.36	0.72	−0.31	0.21
≥40	−0.53	0.14	−3.73	<0.001	−0.81	−0.25
**Marital status**						
Unmarried/widowed/divorced/separated^*^	0.00	–	–	–	–	–
Married	0.37	0.11	3.44	<0.001	0.16	0.59
**Educational level**						
Junior college degree or less^*^	0.00	–	–	–	–	–
Bachelor degree and above	0.46	0.10	4.34	<0.001	0.25	0.66
**Technical title**						
Junior^*^	0.00	–	–	–	–	–
Intermediate	0.61	0.09	6.51	<0.001	0.43	0.80
Senior	0.68	0.15	4.64	<0.001	0.39	0.96
**Type of hospital**						
Emergency center^*^	0.00	–	–	–	–	–
Primary	0.30	0.16	1.86	0.06	−0.02	0.61
Secondary	0.33	0.10	3.23	0.001	0.13	0.54
Tertiary	0.38	0.11	3.45	<0.001	0.16	0.59
**Monthly income (CNY)**						
≤4000^*^	0.00	–	–	–	–	–
4001~6000	−0.17	0.09	−2.01	0.04	−0.34	−0.004
≥6001	−0.45	0.09	−4.75	<0.001	−0.63	−0.26
**Frequency of night shift (per month)**						
0~5^*^	0.00	–	–	–	–	–
6~10	0.43	0.10	4.42	<0.001	0.24	0.62
≥11	1.18	0.11	10.72	<0.001	0.97	1.40
**Self-perceived shortage of physicians**						
No^*^	0.00	–	–	–	–	–
Yes	2.22	0.08	26.86	<0.001	2.06	2.38
**Verbal abuse in the workplace**						
No^*^	0.00	–	–	–	–	–
Yes	1.48	0.10	15.05	<0.001	1.29	1.67
**Physical violence in the workplace**						
No^*^	0.00	–	–	–	–	–
Yes	0.84	0.08	10.04	<0.001	0.67	1.00

* Reference; R^2^ = 0.170; F = 119.10, P < 0.001.

## Discussion

This study investigated the work-family conflict and related factors of emergency department physicians in China. The results showed that ~70% of emergency department physicians were in the high work-family conflict group, which is higher than that of French emergency department physicians (50.1%) (4). It may be attributed to the differences in the emergency department working environment in different countries. A previous report revealed that the average annual income of Chinese physicians was lower than that of developed countries (25), and our results indicated that participants with high monthly incomes had lower scores of work-family conflict.

Gender difference of work-family conflict has always been a concern in the world (13, 26). This study revealed that male emergency department physicians had significantly higher work-family conflict scores than females. However, in Japan, females were reported to be more easily to experience work-family conflict (26). This may be caused by cultural differences in different countries and regions. In traditional Chinese social culture, men, as the primary breadwinners, are asked to dedicate more time and energy to work (27). At the same time, they are allowed to take on less responsibility in the home (28). However, with the increase in dual-earner families, a new fathering ideal has emerged in recent years in which fathers are expected to be involved in child care and domestic responsibilities (29). Because men are expected to not only take responsibility for raising a family, but also share care work with their partners at home, they are more likely to experience work-family conflict in China nowadays.

Our findings showed that emergency department physicians over 40 years old had a lower work-family conflict. This may be due to the fact that most participants in this age group were in a relative balance of work and family (30). They are more capable of dealing with the role conflict between the two fields. Besides, married emergency department physicians scored higher on work-family conflict than physicians in single or other marital status, which is consistent with the previous study (21). The probable reason may be that the married ones have more opportunities to share family responsibilities, such as parenting and doing housework (31). Hospital administrators should pay more attention to aged <40 years old and married physicians on the issue of work-family conflict.

In terms of work-related factors, emergency department physicians with higher educational level and technical title had higher work-family conflict scores. As we all know, these physicians have accumulated more medical knowledge and professional skills, and they undertake heavier emergency tasks in department (32). Their work takes up a greater proportion of time and is prone to conflict with their family roles (33). Therefore, the work-family conflict of emergency department physicians with highly educated and higher professional titles also needs extra attention.

Regarding to the hospital environment, the type of hospitals significantly associated with emergency department physicians' work-family conflict. The more serious the work-family conflict faced by physicians working in high-level hospitals, except in primary hospitals. It is reported that the number of hospital visits in descending order in China was tertiary hospital (1,854.79 million), secondary hospital (1,284.93 million), primary hospital (224.64 million), and other hospitals (213.01 million) (34). Therefore, physicians in high-level hospital are more easily to suffer from time-conflict between work role and family role. Moreover, physicians who experienced workplace violence, whether verbal abuse or physical violence, had higher scores on work-family conflict in this study. This may be because workplace violence can increase the psychological strain of emergency department physicians and negatively influence their family life with partners (35). It is recommended to develop friendly workplace policies for emergency department physicians, especially for tertiary hospitals.

This study also revealed that variables reflecting workload, such as the frequency of night shifts and self-perceived shortage of physicians in department, were significantly associated with work-family conflict of emergency department physicians. Participants with a high frequency of night shifts were more likely to experience work-family conflict, which was consistent with previous studies (36, 37). This is because more night shifts per month mean more time spent at work, which inevitably conflicts with family obligations. In addition, long-term irregular work schedules can affect physicians' moods, which in turn affects their family life (38, 39). In addition, respondents who perceived a shortage of emergency department physicians experienced a higher level of work-family conflict. The possible reason could be that a shortage of physicians leads to an increased workload for the physician on staff. As the work takes up more and more time and energy, it will interfere with the emergency department physicians' family life (23). Therefore, hospital administrators are suggested to establish a scientific shift system and keep the number of emergency department physicians to meet work demands.

### Strengths and limitations

This is the first nationwide study to explore the current situation and related factors of work-family conflict among emergency department physicians in China. What's more, the work-related factors identified in this study are of importance in reducing work-family conflict among emergency department physicians. However, there are still some limitations. First, this was a cross-sectional study, which is limited in establishing a causal relationship between dependent and independent variables. Prospective studies are needed in further studies. Second, this study was conducted in China, and thus, the generalizability of our conclusion to other countries may be limited. Third, there are possibly more factors associated with work-family conflict among emergency physicians than explored in this study; therefore, we could not explore them all.

## Conclusion

Most emergency department physicians experience high levels of work-family conflict in China. Hospital administrations should pay more attention to emergency department physicians who are male, younger than 40 years, married, highly educated, highly titled, working in a high-level hospital, earning <4,000 CNY per month, working a high number of night shifts, perceived understaffing, and experiencing verbal abuse and physical violence in the workplace. To reduce work-family conflict in the emergency department physicians, hospital administrators should develop family-friendly workplace policies, like job sharing, maternity or paternity leave, and parental leave, establish a scientific shift system, and keep the number of physicians to meet work demands.

## Data availability statement

The raw data supporting the conclusions of this article will be made available by the authors, without undue reservation.

## Ethics statement

The studies involving human participants were reviewed and approved by Research Ethics Committee in Hainan Medical University. The patients/participants provided their written informed consent to participate in this study.

## Author contributions

SJY, CJL, JWZ, and NJ were responsible for the conception, design, and writing of the manuscript. JLZ, YFW, and MGT were responsible for the acquisition of data and literature research. NJ, CJL, LL, and XZ were responsible for the analysis and interpretation of data. All authors read and approved the final manuscript.
