# Supplementary material for: Work-family conflict and its related factors among emergency department physicians in China: A national cross-sectional study
Source: Front Public Health. 2023 Mar 20;11:1092025. doi: 10.3389/fpubh.2023.1092025 (PMC10067613; doi:10.3389/fpubh.2023.1092025)
Supplement: Supplementary file 1 [file Table_1.DOCX]

Table S1. Correlation between the independent variables.

|  | 1 | 2 | 3 | 4 | 5 | 6 | 7 | 8 | 9 | 10 | 11 | 12 |
| --- | --- | --- | --- | --- | --- | --- | --- | --- | --- | --- | --- | --- |
| 1. Gender | 1.0000 |  |  |  |  |  |  |  |  |  |  |  |
| 2. Age | -0.0621^*^ | 1.0000 |  |  |  |  |  |  |  |  |  |  |
| 3. Educational level | 0.0216^*^ | -0.1501^*^ | 1.0000 |  |  |  |  |  |  |  |  |  |
| 4. Marital status | -0.0736^*^ | 0.3697^*^ | -0.0067 | 1.0000 |  |  |  |  |  |  |  |  |
| 5. Technical title | -0.0006 | 0.6353^*^ | 0.1142^*^ | 0.2585^*^ | 1.0000 |  |  |  |  |  |  |  |
| 6. Type of hospital | 0.0025 | -0.0643^*^ | 0.2066^*^ | -0.0056 | 0.0967^*^ | 1.0000 |  |  |  |  |  |  |
| 7. Monthly income | -0.0286^*^ | 0.1617^*^ | 0.1546^*^ | 0.0695^*^ | 0.2931^*^ | 0.2314^*^ | 1.0000 |  |  |  |  |  |
| 8. Years of service | -0.1055^*^ | 0.5162^*^ | -0.0090 | 0.2598^*^ | 0.4514^*^ | -0.0007 | 0.2061^*^ | 1.0000 |  |  |  |  |
| 9. Frequency of night shift | -0.1196^*^ | -0.0645^*^ | -0.0060 | 0.0058 | -0.1193^*^ | 0.0004 | -0.0401^*^ | 0.0329^*^ | 1.0000 |  |  |  |
| 10. Self-perceived shortage of physicians | -0.0654^*^ | 0.1101^*^ | 0.0557^*^ | 0.0825^*^ | 0.0996^*^ | 0.0197^*^ | 0.0484^*^ | 0.1368^*^ | 0.1353^*^ | 1.0000 |  |  |
| 11. Verbal abuse | -0.1078^*^ | 0.0502^*^ | 0.1097^*^ | 0.0490^*^ | 0.0737^*^ | 0.0748^*^ | 0.0619^*^ | 0.1198^*^ | 0.1555^*^ | 0.2111^*^ | 1.0000 |  |
| 12. Physical violence | -0.1669^*^ | 0.03771^*^ | 0.0438^*^ | 0.0183 | 0.0315^*^ | -0.0114 | -0.0381^*^ | 0.0998^*^ | 0.1489^*^ | 0.1237^*^ | 0.2642^*^ | 1.0000 |

^*^*P*<0.05
